# Analysis of VEGF, IGF1/2 and the Long Noncoding RNA (lncRNA) H19 Expression in Polish Women with Endometriosis

**DOI:** 10.3390/ijms25105271

**Published:** 2024-05-12

**Authors:** Beata Smolarz, Tomasz Szaflik, Hanna Romanowicz, Magdalena Bryś, Ewa Forma, Krzysztof Szyłło

**Affiliations:** 1 Laboratory of Cancer Genetics, Department of Pathology, Polish Mother’s Memorial Hospital Research Institute, Rzgowska 281/289, 93-338 Lodz, Poland; hanna-romanowicz@wp.pl; 2 Department of Gynecology, Oncological Gynecology and Endometriosis Treatment, Polish Mother’s Memorial Hospital Research Institute, 93-338 Rzgowska-Lodz, Poland; tomaszszaflik@gmail.com (T.S.); kszyllo@o2.pl (K.S.); 3 Department of Cytobiochemistry, Faculty of Biology and Environmental Protection, University of Lodz, Pomorska 141/143, 90-237 Lodz, Poland; magdalena.brys@biol.uni.lodz.pl (M.B.); ewa.forma@biol.uni.lodz.pl (E.F.)

**Keywords:** endometriosis, VEGF, IGF1/2, H19, RT-PCR

## Abstract

The coordinated action of VEGF, IGF1/2 and H19 factors influences the development of endometriosis. The aim of this study was to analyze the expression level of these genes in patients with endometriosis. The study group consisted of 100 patients who were diagnosed with endometriosis on laparoscopic and pathological examination. The control group consisted of 100 patients who were found to be free of endometriosis during the surgical procedure and whose eutopic endometrium wasnormal on histopathological examination. These patients were operated on for uterine fibroids. Gene expression was determined by RT-PCR. The expression of the *VEGF* gene was significantly higher in the samples classified as clinical stage 1–2 compared to the control material (*p* < 0.05). There was also a statistically significant difference between the samples studied at clinical stages 1–2 and 3–4 (*p* < 0.01). The expression of the *VEGF* gene in the group classified as 1–2 was significantly higher. *IGF1* gene expression was significantly lower both in the group of samples classified as clinical stages 1–2 and 3–4 compared to the control group (*p* < 0.05 in both cases). The expression of the *H19* gene was significantly lower in the group of samples classified as clinical stage 3–4 compared to the control group (*p* < 0.01). The reported studies suggest significant roles of *VEGF*, *IGF* and *H19* expression in the pathogenesis of endometriosis.

## 1. Introduction

**Figure 1:**
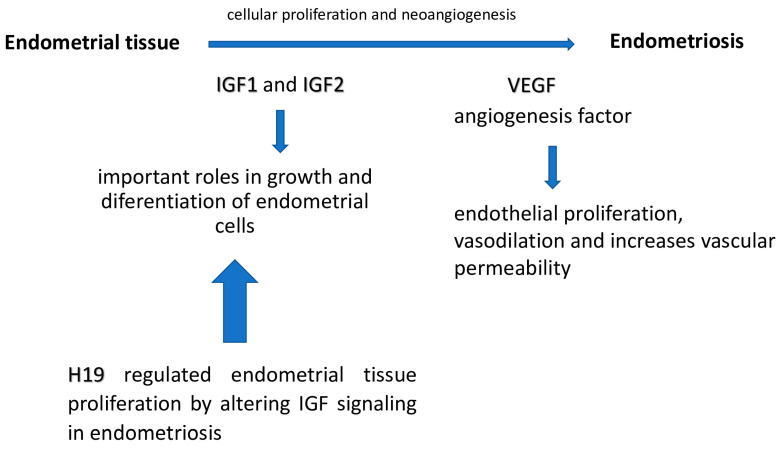
The roles of VEGF, IGF1/2 and H19 in endometriosis.

Endometriosis is a hormone-dependent, chronic and progressive disease characterized by the presence of functioning endometrial tissue outside the uterine cavity [1,2]. Key to the pathogenesis of endometriosis are endometrial cell adhesion and proliferation, as well as cellular invasion and neoangiogenesis (Figure 1) [3].

Therefore, growth factors such as insulin-like growth factors (IGF1 and IGF2), which are produced by endometrial tissue, may play a role in inducing cell proliferation and differentiation [4]. The highest levels of IGF1 and IGF2 expression occur in the late and early proliferative phases, respectively [5]. Angiogenesis is an important process in the development of endometrial tissue, regulated by vascular endothelial growth factor (VEGF) [6]. The activity of VEGF is not limited only to vascular endothelial cells, where it stimulates mitosis and their migration. It has been proven that this protein also affects the migration of monocytes and macrophages and increases capillary permeability (hence the name: vascular permeability factor) [7]. Patients with endometriosis have increased concentrations of VEGF in the peritoneal fluid compared to patients without endometriosis [8]. Long non-coding RNAs (lncRNAs) are numerous non-protein-coding transcripts longer than 200 nucleotides [9]. It has been proven that changes in the expression of these molecules lead to changes in gene expression in cancer cells [10]. LncRNA H19 (H19) was the first lncRNA to function as an oncogene in many malignant tumors [11]. In addition to H19’s established role in promoting cell growth, proliferation, invasion, migration, epithelial–mesenchymal transition (EMT) and metastasis, H19 has recently been discovered to inhibit programmed cell death (PCD) of cancer cells [12].Endometriosis is not classified as a malignant neoplasm. Nevertheless, due to its complex structure, which includes stromal, epithelial and vascular components, as well as due to changes in the genetic material, the structure of endometriosis cells is similar to thatobserved in malignant tumors. In addition, in the endometriosis, the percentage of abnormal (ectopic) cells is much higher than in the normal endometrium. Previous studies have not clearly shown an increased risk of cancer transformation in patients with endometriosis, but they have shown that this group of patients is more likely to have ovarian cancer, non-Hodgkin lymphomas and other cancers such as colorectal cancer, thyroid cancer or melanoma [13]. The development of endometriosis is possible through the action of H19, which modulates the EMT process [14]. Studies have shown that H19 expression is extinguished in mononuclear cells obtained from the peritoneal fluid of endometriosis patients [15]. As a result of activation of the H19/miR-216a-5p/ACTA2 pathway, the formation of fibrotic tissue is stimulated in patients with endometriosis [16]. In in vivo experiments on naked mice, H19 silencing led to a significant reduction in the incidence of endometriosis [17]. In endometriosis foci, increased expression of cyclooxygenase-2 (COX-2) has been observed at the same time, which may lead to increased production of prostaglandin E2 (PGE2). Interestingly, COX-2 expression has also been shown to be stimulated by IL-1β, VEGF, PGE2 and estradiol acting through the type β receptor. 17β-estradiol is known to play a role in regulating H19 expression and function in endometriosis patients. 17β-estradiol is therefore involved in the development of endometriosis by regulating H19 [18]. H19 regulates endometrial tissue proliferation by altering IGF signaling in endometriosis [19]. Studies have shown reduced levels of H19 expression in ectopic and eutopic endometrial tissue compared to controls. It is hypothesized that H19, through downregulation of IGF1 and IGF2, leads to impaired regulation of cell growth and differentiation [20]. Reduced expression of H19 has been shown in the endometrial tissues of infertile women [21].

Most of the research on endometriosis comes from US, UK, Canadian and Australian data. So far, there has beenno broader study on the Polish area; hence, the generally available data may differ from those carried out in the American or British populations. The ethnic factor plays a major role in the incidence of endometriosis [22]. Currently available data show a higher incidence of endometriosis among white women compared to black women. Other data indicate that the incidence of endometriosis among Japanese women is twice as high compared to Caucasians. Similarly, women from Southeast Asia have a higher incidence of endometriosis.

Women from Iran were tested for the expression of the *VEGF*, *IGF1/2* and *H19* genes [23]. It was noted that the expression profiles of the *H19*, *IGF1* and *IGF2* genes werereduced in eutopic and ectopic endometrial tissues of patients with endometriosis compared to control tissues. *VEGF* gene expression was increased in eutopic tissues compared to controls. In contrast, its expression level was lower in ectopic lesions compared to eutopiclesions and controls. The work of scientists from Iran has shown that the expression of the *VEGF*, *IGF1*, *IGF2* and *H19* lncRNA genes and the epigenetic changes of H19 lncRNA play a dynamic role in the pathogenesis of endometriosis [23]. However, the Iranian study included a small group of 24 patients.

The interesting results of the study led us to conduct similar analyses on a larger study group of a different population. Current data indicate that the incidence of endometriosis may vary from population to population. The patients in our study were from the Polish population. The aim of the study was to analyze the expression levels of the *VEGF*, *IGF1/2* and *H19* genes in patients with endometriosis and in the control group. The obtained results were correlated with clinical–pathological data in order to determine their significance for the risk of endometriosis.

## 2. Results

### Expression Analysis of the VEGF, IGF1, IGF2 and H19 Genes

**Figure 2:**
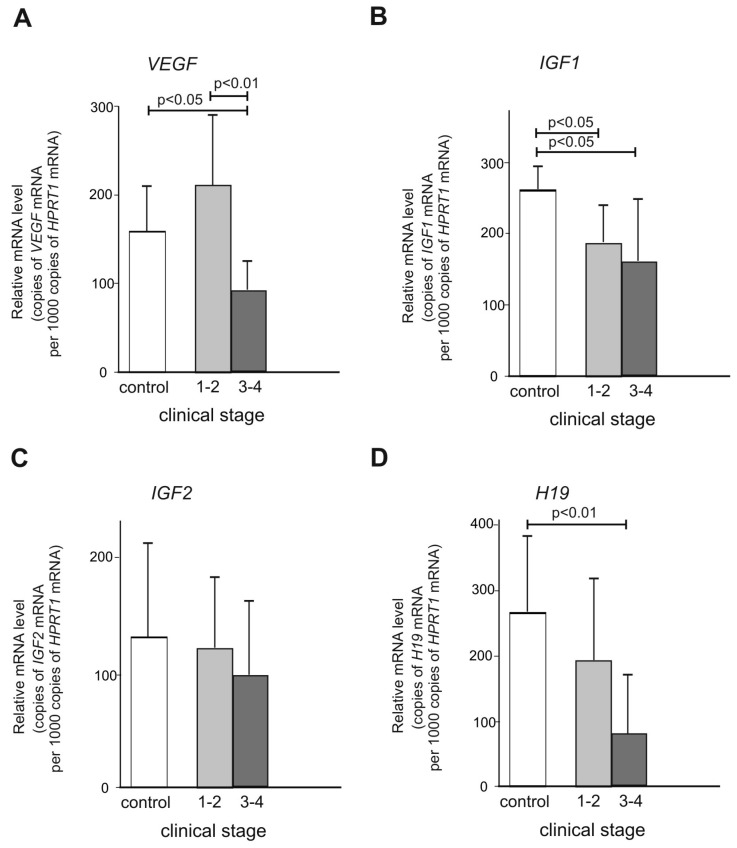
Relative mRNA expression levels of *VEGF* (**A**)*, IGF1* (**B**)*, IGF2* (**C**) and *H19* (**D**) genes in tissue samples (*n* = 100 in total) classified as clinical stage 1–2 and 3–4 obtained from endometriosis patients vs. control group (*n* = 100).Data are presented as mean +/- standard deviation (SD).

The expression of the *VEGF* gene was significantly higher in the samples classified as clinical stage 1–2 compared to the control material (*p* < 0.05). There was also a statistically significant difference between the samples studied at clinical stages 1–2 and 3–4 (*p* < 0.01). The expression of the *VEGF* gene in the group classified as 1–2 was significantly higher (Figure 2A).

*IGF1* gene expression was significantly lower sin the group of samples classified as clinical stages 1–2 and 3–4 compared to the control group (*p* < 0.05 in both cases). There were no statistically significant differences in *IGF1* gene expression between the groups at clinical stages 1–2 and 3–4 (*p* > 0.05) (Figure 2B).

In the case of *IGF2* gene expression, no statistically significant differences were found in the studied material (Figure 2C).

The expression of the *H19* gene was significantly lower in the group of samples classified as clinical stage 3–4 compared to the control material (*p* < 0.01) (Figure 2D).

In the course of further analyses, no statistically significant relationships between the expression of the *VEGF*, *IGF1*, *IGF2* and *H19* genes and other clinical and pathological features (e.g., age, BMI, deliveries and spontaneous abortion) were found (Table 1).

## 3. Discussion

For the development of endometriosis, the proliferation of endometrial tissue and neoangiogenesis are necessary. Insulin-like growth factors 1 and 2 act as inducers of cell proliferation. In contrast, vascular endothelial growth factor is an inducer of angiogenesis. lncRNA H19 may play a role in the pathogenesis of some diseases, as it regulates gene expression through epigenetic mechanisms by interacting with chromatin-modifying complexes. A close relationship between the H19 and IGF1 signaling pathways has been demonstrated [24,25]. LncRNA H19 can regulate the IGF-1 signaling pathway and, consequently, the proliferation and apoptosis of endometrial stromal cells [26]. H19 binds the methyl-cpG binding domain protein 1 (MBD1). The H19–MBD1 complex binds to methylated DNA, after which it recruits histone lysine methyltransferases (KMTs) to silence these genes by chromatin thickening (H3K9 methylation). A number of studies have observed the effect of H19-DMR (differentially methylated region)methylation on IGF2 expression, resulting from a loss of IGF2 imprinting [27,28].Recent studies have shown that modifications to the methylation of H19-DMR region II can alter the level of IGF2 expression, but have no effect on H19 expression [23]. It is hypothesized that epigenetic modifications of the remaining H19-DMR regions may possibly affect the expression profile of the H19 gene.

Angiogenesis is one of the key processes responsible for the implantation and growth of endometriotic lesions and the formation of adhesions [29]. Angiogenesis is regulated by the activity of pro- and antiangiogenic factors. Factors that stimulate angiogenesis include VEGF [30]. Hypoxia-stimulated VEGF is one of the most potent mediators of angiogenesis, and is responsible for most of its stages. Studies have shown increased concentrations of VEGF in the peritoneal fluid, serum and ectopic endometrium from women with endometriosis [31,32]. Endometriosis is an estrogen-dependent disease. Studies have shown that estrogen increases the regulation of cytoskeletal genes and proteins (CK-18, TGF-β, TNF)-α) and VEGF in the peritoneum, which affects the process of angiogenesis, cell proliferation, fibrosis and inflammatory markers. This can lead to the development of endometriosis [33].In the presented studies, *VEGF* expression was higher in stage 1–2 patients compared to controls. Similar differences were found in the stage 1–2 versus stage 3–4 groups. *VEGF* overexpression in stage 1–2 endometriosismay indicate higher angiogenic activity, which may contribute to increasing the possibility of endometrial cell implantation in ectopic places. The results of previous studies, consistent with this one, have shown that *VEGF* expression in the endometrial tissue and peritoneal fluid of endometriosis patients is increased compared to controls, although it does not differ significantly between different stages of the disease [34,35]. In one divergent study, the concentration of *VEGF* in the peritoneal fluid between endometriosis patients and healthy controls did not differ significantly [36]. Other analyses showed that gene expression of *VEGF* receptors was also higher in ectopic endometrial lesions than in eutopic tissue [37]. The results of the latest work showed that *VEGF* expression in the ectopic endometrium was significantly higher compared to the eutopic endometrium and control [38]. The *VEGF* level increases in theeutopic and ectopic endometrium of patients with endometriosis [37,39]. 

IGF-1 is known to be one of the factors that prevents apoptosis and acts as a mitogen on endometrial cells, causing them to grow in the peritoneal cavity [40,41]. Macrophage-derived IGF-1 may be a key factor involved in the production of pain in endometriosis [42]. A study by Blontzos et al. indicates that different forms of endometriosis can develop indifferent molecular pathways. They conducted interesting studies regarding thedifferential expression of insulin growth factor 1 isoforms in deeply infiltrating endometrial lesions (deep-infiltrating endometriosis (DIE)), in ovarian endometriosis and in the eutopic endometrium of the same endometriosis patients. The results were compared with expression in the eutopic endometrium of women without endometriosis. Lower expression of IGF-1Ea and IGF-1Ec was found in endometrial tumors without DIE compared to endometriosis with concomitant DIE or in DIE nodules [43]. In the presented studies, *IGF1* expression was lower in stage 1–2 patients compared to controls. Similar differences were found in thestage1–2 versus stage 3–4 groups. In the case of *IGF2*, no differences were detected in the test material. Our research is partially different from the work of other researchers. Heidari et al. report that higher concentrations of *IGF1* in endometrial ectopic cells in patients with endometriosis may contribute to the development of endometriosis [44]. Another study showed a decrease in *IGF2* expression levels in eutopic and ectopic endometriosis endometrial cells compared to controls. *IGF1* levels were reduced in the eutopic endometrium in endometriosis compared to control endometrial samples, while increased *IGF1* expression was observed in fibrotic peritoneal adhesions [45]. A significant reduction in *IGF1* expression in cystic endometriosis was observed compared to eutopic endometriosis in women with endometriosis [46]. Our results were inconsistent with another study showing significantly higher expression of *IGF1* in ectopic endometrial stromal cells compared to eutopic cells and controls [47]. 

H19 may act as a tumor suppressor, keeping cells from growing and dividing too quicklyor in an uncontrolled way. Studies indicate that the knockdown of lncRNA H19 inhibits tumor formation in vivo [48,49]. Endometriosis is not classified as a malignant neoplasm; however, its complex structure (epithelial, stromal and vascular components) and changes in genetic material (loss of hetrozygosity of tumor suppressor genes) are similar to those observed in malignant tumors. Also, the percentage of abnormal cells in the ectopic endometrium is much higher than in the normal endometrium. Banz et al., using the microarray technique, showed differences in gene expression in endometrial carcinoma tissue and endometroid carcinoma arising in the scopeof endometriosis [50]. H19 is involved in the pathogenesis of endometriosis by modulating the proliferation and invasion of ectopic endometrial cells [18]. In a study by Liu et al. lncRNA H19 showed the greatest upregulation in the ectopic and eutopic endometrium [51]. In further studies, it turned out that lowering the level of lncRNA H19 as a result of modulation of miR-124-3p and ITGB3 in vitro can inhibit ectopic proliferation and invasion of endometrial cells [52]. In vivo studies have shown that the knockdown of lncRNA H19 inhibits endometriosis in naked mice [17].lncRNA H19, which acts as a molecular sponge, reduces the bioavailability of let-7 microRNAs [53]. As a result of binding to complementary sequences in mRNA, let-7 inhibits the expression of the target gene. As a consequence, translational repression and mRNA degradation occur [54]. Among the genes that target let-7 is IGF1R(Insulin-Like Growth Factor 1 Receptor) [54]. Due to the fact that women with unexplained infertility show reduced expression of *H19* in the eutopic endometrium, *H19* regulates let-7 and let-7 targets IGF1R, and it is useful to determine the role of *H19* in the endometrium and its relationship to IGF signaling in endometriosis [54,55]. The results of some studies have shown reduced *H19* expression in endometriosis patients to be correlated with reduced levels of *IGF1R* mRNA in the eutopic endometrium compared to women without endometriosis [19]. This is the result of the regulation of IGF1R by H19, although IGF1R may be regulated by mechanisms other than H19.

In our study, we have shownthat the expression of both *H19* and *IGF1* is significantly reduced in women with endometriosis. This may be due to the fact that reduced *H19* expression leads to increased let-7 bioavailability, which in turn inhibits *IGF1R* expression at the post-transcriptional level, thus contributing to reduced stromal cell proliferation. Disruption of this newly identified *H19/Let-7/IGF1R* regulatory pathway may contribute to impaired endometrial preparation and receptivity in women with endometriosis.In our opinion, the presented work is the first study in which the expressions of *VEGF, IGF1/2* and *H19* have beencorrelated with clinical–pathological factors. We did not find any such works in the PubMed database. In our study, we found no statistically significant relationships between the expression of *VEGF, IGF1, IGF2* and *H19* genes and other clinical and pathological features, such as age, BMI, deliveries or spontaneous abortions.

The results presented in this study concerning the analysis of *VEGF, IGF1/2* and *H19* genes show the presence of certain dependencies of their expression with endometriosis. We would like to emphasize that the presented studies cover a small population. Therefore, they require further work on much larger groups of respondents in order to draw the right conclusions. Beyond that, there is still little known about the role of this “transcriptional noise” and how to thoughtfully design a study to draw definitive and defensible conclusions. Moreover, a limitation of our study is the lack of proteomic analysis including VEGF and IGF1/2 proteins. Results obtained by methods such as Western blotting or immunohistochemistry would add value to the work. Conclusions based on both measurements of mRNA and protein expression in tissues would be more convincing than qPCR analysis alone, and could provide more benefits for the future diagnosis and personalized medicine of endometriosis.Bearing in mind all the above findings and being aware of the limitations of our study, we dare to claim that the results obtained in this paper contribute to the expansion of knowledge regardingthe molecular mechanisms that promote the development of endometriosis. The study showed differences in the expression of the studied genes between the studied groups of patients, which may potentially promote the development of the disease. Due to the controversial results of studies on gene expression analyses in patients with endometriosis, the result of the study makes an important contribution to expanding knowledge about the influence of genetic factors on the development of endometriosis. Gene expression testing could be a target for personalized therapy in the future.

## 4. Materials and Methods

### 4.1. Patients

Tissue specimens collected from patients with endometriosis (*n* = 100) and control patients (*n* = 100) and embedded in paraffin blocks provided the material for our studies. The paraffin blocks were provided by the Archive of the Department of Clinical Pathomorphology, Polish Mother’s Memorial Hospital Research Institute in Lodz, Poland. The study group comprised 100 patients who underwent surgery for endometriosis at the Department of Gynecology, Oncological Gynaecology and Endometriosis Treatment of the Polish Mother’s Memorial Hospital Research Institute in Lodz during the years 2015–2016. Endometriosis was confirmed by intraoperative diagnostics and, finally, by histopathological evaluation. The characteristics of the patients are presented in Table 2. Histopathological assessments confirmed normal endometrium in all the controls. The control patients did not suffer from endometriosis. These patients were operated on for uterine fibroids.

Representative specimens from the lesions of the operated patients were immediately sent to the pathology laboratory. The sliceswere frozen in a cryostat (with freezing temperatures lower than −20 °C).The frozen material was cut into very thin sections to create a microscopic preparation.Histopathological examination was carried out by a pathomorphologist whose aim was to make a pathomorphological diagnosis (determination of the nature of the lesion).The tissues were then placed in a concentrated ethanol solution and fixed in liquid paraffin. The tissue fragments embedded in paraffin hardened and took the form of paraffin blocks. Such material is very durable and can be tested many times, even several years after its preparation.

The clinical stages of endometriosis were determined according to rASRM (The Revised American Society for Reproductive Medicine classification of endometriosis, 1996) [56]. Formal consent (No. 88/2022) was obtained from the Bioethics Committee at the Polish Mother’s Memorial Hospital Research Institute in Lodz, Poland. Gene expression studies were carried out at the Department of Cytobiochemistry, Faculty of Biology and Environmental Protection, University of Lodz, Pomorska 141/143, 90-237 Lodz, Poland.

### 4.2. RNA Isolation

Paraffin was removed from the samples from which RNA was isolated by successive rinsing of the samples in xylene (4–8 times) and then in ethanol (99.8%, 3–4 times). The tissue (after paraffin removal) was dried and homogenized in 850 μL of denaturing buffer (4 M guanidine isothiocyanide, 0.25 M sodium citrate, 0.5% sarcosyl, 0.1 M mercaptoethanol) with the addition of 250 μL proteinase K solution (20 mg/mL),then incubated at 55 °C overnight (16–18 h). After incubation, the sample was centrifuged (14,000× *g*, 5 min, 4 °C). After centrifugation, 1 mL of Trizol reagent (Ambion, Austin, TX, USA) was added to the resulting supernatant (200 μL).

The subsequent stages of the procedure were carried out in accordance with the manufacturer’s instructions. The resulting mixture was extracted with 0.2 mL chloroform. After extraction, the aqueous phase was precipitated by the addition of 0.5 mL isopropanol. It was centrifuged (12,000× *g*, 10 min, 4 °C), and the resulting RNA precipitate was washed with 75% ethyl alcohol. The precipitate was dried and dissolved in 100 μL of TE buffer. The RNA was precipitated again overnight at −70 °C using the following mixture: 220 μL 99.8% ethyl alcohol and 10 μL 3 M sodium acetate. The whole mixture was centrifuged (12,000× *g*, 10 min, 4 °C). The pellet was dried and dissolved in water (20–100 μL).RNA samples were stored at a temperature of −20 °C. Isolated RNA was usually stored for no more than 24–48 h. This time did not affect the quality or integrity of the RNA. RNA concentrations were measured by the spectrophotometric method, as previously described [57,58].

### 4.3. Quantitative Real Time Polymerase Chain Reaction (qRT-PCR)

Two-step qRT-PCR was used to analyze the expression of *VEGF*, *IGF1*, *IGF2* and *H19* genes in normal tissue and endometriosis samples. The High-Capacity cDNA Synthesis Kit (ThermoFisher, Waltham, MA, USA) was used for the cDNA synthesis. Quantitative analysis of the studied mRNAs was performed using an Eppendorf Matercyclerrealplex 4 instrument (Eppednorf, Hamburg, Germany) with HOT FIREPol^®^EvaGreen qPCR Supermix reagent (Solis BioDyne, Tartu, Estonia) with the following PCR protocol: 95 °C for 15 s, 60 °C for 30 s and 72 °C for 30 s. Ct values were used for subsequent analysis, and HPRT1 was used as the endogenous control. The primer sequences are listed in Table 3.

### 4.4. Statistical Analysis

The obtained results were statistically processed by means of the PQStat v. 1.6.6 (PQStat Software, Poznan, Poland). The significance of differences was analyzed at the level of gene expression and mRNA using non-parametric tests (the *U* Mann–Whitney test and the Kruskal–Wallis test) for a lack of distribution normality of the obtained results, as confirmed by the Shapiro–Wilk test. The Spearman rank-order correlation coefficient test was used to assess correlations between variables. Statistical significance was confirmed at *p* < 0.05.

## 5. Conclusions

Elevated levels of *VEGF* expressioncorrelate with decreasedlevels of *IGF1* and *H19* in endometriosis. Elevated levels of *VEGF* expression in stage 1–2 of endometriosis maylead to additional implantation of endometrial fragments in the uterine cavity. Decreased *H19* expression in endometriosislesions likely decreases *IGF1* expression. This associations suggests that the cells of endometriotic tissue possibly undergo an impairment of cellular growth regulation and differentiation.

## Figures and Tables

**Table 1:** Correlation between clinical and pathological features and relative gene expression.

Patients (*n* = 100)	Control (*n* = 100)
Spearman’s rank correlation	
Age	
VEGF	
r = 0.11, *p* = 0.24	r = 0.32, *p* = 0.47
IGF1	
r = 0.08, *p* = 0.33	r = 0.15, *p* = 0.28
IGF2	
r = −0.16, *p* = 0.71	r = 0.31, *p* = 0.49
H19	
r = −0.61, *p* = 0.018	r = −0.12, *p* = 0.41
BMI	
VEGF	
r = 0.52, *p* = 0.19	r = 0.15, *p* = 0.33
IGF1	
r = 0.53, *p* = 0.18	r = 0.28, *p* = 0.09
IGF2	
r = 0.83, *p* = 0.13	r = 0.73, *p* = 0.23
H19	
r = 0.32, *p* = 0.67	r = 0.48, *p* = 0.11
**Kruskal–Wallis test**	
Deliveries	
*VEGF*	
*p* = 0.1572	
C0 vs. C1 C1 vs. C ≥ 2 C0 vs. C ≥ 2 P0 vs. P1 P0 vs. P ≥ 2 P1 vs. P ≥ 2	ns ns ns ns ns ns
*IGF1*	
*p* = 0.071	
C0 vs. C1 C1 vs. C ≥ 2 C0 vs. C ≥ 2 P0 vs. P1 P0 vs. P ≥ 2 P1 vs. P ≥ 2	ns ns ns ns ns ns
*IGF2*	
*p* = 0.35	
C0 vs. C1 C1 vs. C ≥ 2 C0 vs. C ≥ 2 P0 vs. P1 P0 vs. P ≥ 2 P1 vs. P ≥ 2	ns ns ns ns ns ns
*H19*	
*p* = 0.09	
C0 vs. C1 C1 vs. C ≥ 2 C0 vs. C ≥ 2 P0 vs. P1 P0 vs. P ≥ 2 P1 vs. P ≥ 2	ns ns ns ns ns ns
The *U* Mann–Whitney test	
*VEGF*	
C yes vs. C no	*p* = 0.39
P yes vs. P no	*p* = 0.18
*IGF1*	
C yes vs. C no	*p* = 0.56
P yes vs. P no	*p* = 0.08
*IGF2*	
C yes vs. C no	*p* = 0.17
P yes vs. P no	*p* = 0.36
*H19*	
C yes vs. C no	*p* = 0.11
P yes vs. P no	*p* = 0.15

C—control; P—patients; ns—not significant.

**Table 2:** Clinical–pathological characteristics of the study groups.

Patients (*n* = 100)		Control (*n* = 100)	
Age (Range) 21–53 Years Age (Mean) 34.89 ± 7.49		Age (Range) 26–67 Years Age (Mean) 37.93 ± 6.01	
BMI, *n* (%) <25 kg/m^2^ 25 ≤ BMI < 30 kg/m^2^ ≥30 kg/m^2^	The number (%) 68 (68%) 22 (22%) 10 (10%)	BMI, *n* (%) <25 kg/m^2^ 25 ≤ BMI < 30 kg/m^2^ ≥30 kg/m^2^	The number (%) 29 (29%) 43 (43%) 28 (28%)
Deliveries 0 1 ≥2	The number (%) 53 (53%) 21 (21%) 26 (26%)	Deliveries 0 1 ≥2	The number (%) 7 (7%) 36 (36%) 57 (57%)
Spontaneous abortion Yes No	The number (%) 5 (5%) 95 (95%)	Spontaneous abortion Yes No	The number (%) 9 (9%) 91 (91%)
Clinical stage I II III IV	The number (%) 26 (26%) 25 (25%) 17 (17%) 32 (32%)		

**Table 3:** Sequences of primer pairs used in this study.

Genes	Primer Sequences (5′–3′)	Product Length (bp)
*VEGF*	F: ACCCACCCACATACATAC	151
	R: CAGCAGTCAAATACATCCAG	
*IGF1*	ATGCTCTTCAGTTCGTGTG	148
	CAATACATCTCCAGCCTCCT	
*IGF2*	F: CCTCTATCCTTG ATACAACAGC	121
	R: AATTCGTCTGATTGTCCAGG	
*H19*	F: GTGACAAGCAGGACATGAC	121
	R: GAAGTAAAGAAACAGACCCGC	
*HPRT1*	F: CCTGGCGTCGTGATTAGTGAT	91
	R: ACACCCTTTCCAAATCCTCAGC	

## Data Availability

All data and materials, as well as software application, support the published claims and comply with field standards.
